# Post-transplant-cyclophosphamide plus everolimus as GvHD prophylaxis in refractory T- and B-cell lymphoma

**DOI:** 10.1038/s41409-024-02472-3

**Published:** 2024-11-15

**Authors:** Tim Richardson, Hishan Tharmaseelan, Lukas Frenzel, Philipp Gödel, Moritz Fürstenau, Pascal Nieper, Till Braun, Daniel Schütte, Michael Hallek, Christof Scheid, Udo Holtick

**Affiliations:** Department I of Internal Medicine, Medical Faculty and University Hospital of Cologne, University of Cologne, Cologne, Germany

**Keywords:** B-cell lymphoma, T-cell lymphoma

## To the Editor:

Aggressive lymphomas are a heterogeneous group with a curative treatment approach. Advances in immunochemotherapy and salvage therapies, including CAR-T cells, have resulted in disease control or cure in up to two-thirds of patients. Allogeneic hematopoietic stem cell transplantation (aHSCT) remains a curative strategy for lymphomas refractory to other treatments. Its success largely depends on factors such as tumor burden, conditioning regimen, and the graft-versus-tumor (GvT) effect [1].

Graft-versus-host disease (GvHD) is a major challenge, with acute GvHD (aGvHD) contributing to early mortality and chronic GvHD (cGvHD) causing late morbidity. Non-relapse mortality (NRM) rates of 10–30% remain common [2].

Traditional immunosuppressive protocols with calcineurin inhibitors (CNI) only prove efficacious in preventing GvHD in a portion of patients, whilst entailing numerous interactions with other medications and posing significant risks of adverse effects, including renal failure, elevated blood pressure, tremors and headaches [3].

Thus, there is a medical need for alternative immunosuppressive strategies.

Post-transplant cyclophosphamide (PTCy) was initially designed to facilitate haploidentical stem cell transplantation. It also offers benefits by preserving a subset of regulatory T-cells (T-regs) responsible for immunosuppressive activity and sparing hematopoietic stem cells [4].

Our OCTET trial showed the potential of PTCy as sole GvHD prophylaxis in matched-donor transplantation, but higher severe aGvHD rates indicated a need for combination strategies [5]. Enhanced PTCy approaches with sirolimus have since shown promise in myeloid malignancies [5, 6].

Everolimus possesses a unique combination of immunosuppressive attributes alongside anti-neoplastic and anti-viral effects. Its effectiveness has been proven in preventing allograft rejection post-solid organ transplantation [7], as well as in the prophylaxis of GvHD and the treatment of cGVHD following aHSCT [8].

Our OCTET-EVER trial underscored the benefits of everolimus, notably its lack of nephrotoxicity and its anti-neoplastic attributes with promising rates of GvHD- and relapse-free survival (GRFS) in this difficult to treat population [9].

Our goal was to confirm the results of the explorative phase II clinical study (Uni-Koeln-1717; EudraCT number: 2013-005507-14) OCTET-EVER. Thirty-three consecutive Lymphoma-patients that were transplanted at our ward between September 2019 and January 2024 were retrospectively evaluable for analysis which was approved by the local Institutional Review Board. We included relapsed and refractory patients with aggressive B-cell and T-cell non-Hodgkin’s lymphoma or Hodgkin’s disease (See supplement for detailed diseases).

Reduced-intensity conditioning (RIC) consisted of 5 days of fludarabine 30 mg/m2 per day accompanied on the first two days by busulfan at 3.2 mg/kg/d given intravenously. PTCy was administered intravenously on day three and four after transplant at 50 mg/kg/ day. Everolimus was given from day +5 to day +100 after transplantation with target blood levels 5–10 ng/ml.

Statistical analysis was performed using IBM SPSS Version 29. Overall survival (OS) and progression-free survival (PFS) were estimated via Kaplan-Meier. NRM and relapse were treated as competing risks. GvHD was assessed according to the 2014 NIH criteria, with aGvHD events defined as grade 3 or higher and cGvHD events requiring systemic immunosuppression.

Thirty-three patients with relapsed and refractory aggressive lymphoma underwent aHSCT. Their characteristics are shown in Table 1. All patients, except those with Richter’s transformation, underwent autologous stem cell transplantation before aHSCT, with a median of four prior therapies. Nine of twelve patients with diffuse large B-cell lymphoma received a CAR-T cell therapy prior to transplant. Median time between CAR-T cells and transplant was 3.8 months.

**Table 1:** Patient and donor characteristics.

	*N* = 33
Age in years, median (range)	54 (22–69)
Female	30,3%
Caucasian	90.1%
Diagnosis	
Hodgkin’s lymphoma	12.1%
Aggressive B-NHL	42.4%
Aggressive T-cell lymphoma	45.5%
Prior lines of therapy, median (range)	4 (2–7)
Time diagnosis to aHSCT, median month	23 (6–156)
Stem cell source, Peripheral Blood	100%
CD34-dose, median, ×10e/6 kg BW	7.0
Donor	
Matched related donor	21.2%
Matched unrelated donor	42.4%
MMUD 9/10 HLA matched	27.3%
Haplo-Transplant	9.1%
Response prior to transplant	
Complete response	9.1%
Partial response	57.6%
Stable / progressive Disease	24.2%
HCT-CI, median (range)	3 (0–8)
CIBMTR Risk group	
Intermediate	78.8%
High	12.1%
Very High	9.1%

Donor engraftment was achieved in all patients. The median time to neutrophil recovery was 17 days (range 13–42), and to platelet recovery, 21 days (range 12–120). Hematological toxicities were similar to the OCETET-EVER data, without new non-hematological safety concerns.

AGvHD occurred in 63.6% of patients, with 33.3% experiencing grade II, and 6,1% patients each experiencing grade III and IV. CGvHD developed in 21.2% of patients, with only 9.1% still on systemic immunosuppression (Ruxolitinib).

**Fig. 1:**
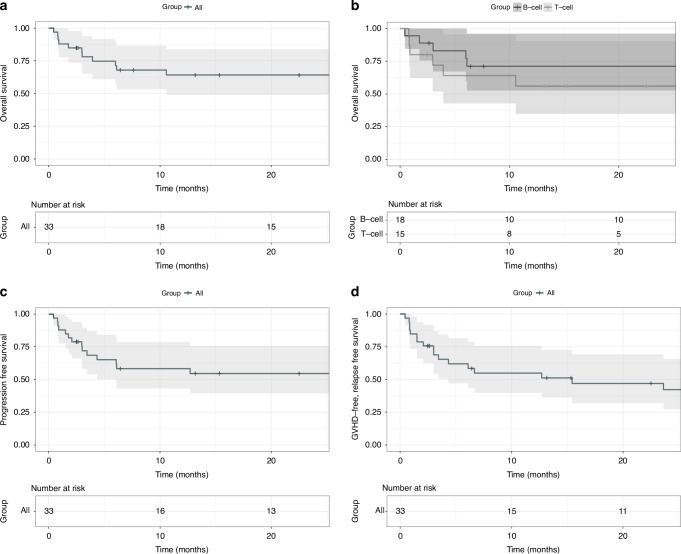
Relevant clinical endpoints. Kaplan–Meier analysis of overall survival (**a**, **b**), progression-free survival (**c**) and GvHD-relapse-free survival (**d**) of all patients (**a**, **c**, **d**)  and divided in B- and T-Cell lymphoma (**b**).

With a median follow-up of 30.8 months, median PFS and OS were not reached. OS was 64% at 1 and 2 years, and PFS was 58% and 55% at year 1 and 2, respectively (Fig. 1a, c). Relapse occurred in 16% and 20% at year 1 and 2, respectively, with a median relapse time of 4 months (range 1.5–12.7). A separate consideration of B- and T-cell lymphomas shows a difference in survival (Fig. 1b) and PFS, though not statistically significant. GRFS was 54% at 1 year and 48% at 2 years (Fig. 1d).

Median time to death for the 13 patients who died was 25.5 months. Five of the six patients with relapse died. NRM was 24.2% at 1 and 2 years. Two patients with COVID-19-related respiratory failure declined intubation. Of the nine patients who received CAR-T cells before transplant, five died, and four remain in remission.

Our findings support the favorable outcome reported in the OCTET-EVER trial, with low GvHD and NRM rates, and a GRFS of 47% and OS of 68% at three years [5]. Our retrospective data show similar results, with a GRFS of 42% and OS of 64% at two years.

The prospective, randomized Hovon-96 trial demonstrated the superiority of PTCy with CsA over CsA with mycophenolic acid but only 24 (25%) of the patients included had aggressive lymphoma. The cumulative incidence of extensive cGvHD was 16% in the PTCy group at two years resulting in a GRFS of 45% [10].

Bolaños-Meade et al. analyzed the efficacy of a PTCy backbone in a randomized phase 3 trial, comparing PTCy, tacrolimus, and MMF to a standard regimen of tacrolimus and methotrexate following a RIC in 8/8 HLA-matched or 7/8 HLA-mismatched transplants. [11]. The GRFS reported was 52,7% at one year for PTCy in combination with tacrolimus and mycophenolic mofetil versus 34,9% for the standard regimen. Most (85%) of these patients had acute leukaemia’s and MDS.

Solomon et al. presented data from a prospective phase 2 trial involving 26 patients with myeloid and lymphoid malignancies treated with PTCy and sirolimus similar to the OCTET-EVER approach. The cumulative incidence of grade III-IV aGvHD and cGvHD was 15%, and 31% with a 2-year OS of 71% [12].

The 2-year NRM of 25% remains the major obstacle and could mainly be attributed to septic complications. NRM was twice as high as compared to the OCTET-EVER trial. One explanation might be the higher disease risk index (DRI) and Hematopoietic-Cell-Transplantation-specific-Comorbidity-Index (HCT-CI) compared to the OCTET-EVER trial population. Additionally, 36,4% of patients did not have a matched donor, while in the trial all patients had matched donors. The lower relapse rate compared to the OCTET-EVER cohort (20% versus 40%) may be explained by the absence of patients with multiple myeloma which demonstrated high relapse rates in the previous trial.

Our retrospective study has limitations, including a small, heterogeneous patient cohort and the lack of a comparator arm. These results need confirmation through prospective randomized trials. In summary, this real-world data highlights the potential of the PTCy/everolimus combination as a viable alternative to CNI-based GvHD prophylaxis in aggressive lymphoma patients undergoing aHSCT.

## Supplementary information

Patient disease entities

## Data Availability

The datasets analyzed in our paper are available from the corresponding author on reasonable request.
