# Supplementary material for: Post-transplant-cyclophosphamide plus everolimus as GvHD prophylaxis in refractory T- and B-cell lymphoma
Source: Bone Marrow Transplant. 2024 Nov 15;60(2):244–6. doi: 10.1038/s41409-024-02472-3 (PMC11810763; doi:10.1038/s41409-024-02472-3)
Supplement: Supplementary file 1 — Patient disease entities [file 41409_2024_2472_MOESM1_ESM.docx]

| ALK negative Anaplastic Large Cell Lymphoma |
| --- |
| ALK negative Anaplastic Large Cell Lymphoma |
| ALK positive anaplastic large cell lymphoma |
| ALK positive anaplastic large cell lymphoma |
| Angioimmunoblastic t-cell lymphoma |
| Angioimmunoblastic t-cell lymphoma |
| Angioimmunoblastic t-cell lymphoma |
| CLL with bone-marrow failure |
| DLBCL |
| DLBCL |
| DLBCL |
| DLBCL |
| DLBCL |
| DLBCL |
| DLBCL |
| DLBCL Richter's Transformation from CLL |
| DLBCL Richter's Transformation from CLL |
| DLBCL Richter's Transformation from CLL |
| DLBCL Richter's Transformation from CLL |
| DLBCL Richter's Transformation from Follicular Lymphoma |
| Extranodal NK/T-cell lymphoma, nasal type |
| Extranodal NK/T-cell lymphoma, nasal type |
| Extranodal NK/T-cell lymphoma, nasal type |
| Folliculotropic mycosis fungoides |
| Hodgkin Lymphoma |
| Hodgkin Lymphoma |
| Hodgkin Lymphoma |
| Hodgkin Lymphoma |
| Monomorphic epitheliotropic intestinal T cell lymphoma |
| Peripheral T-cell lymphoma, not otherwise specified |
| Primary mediastinal B-cell lymphoma |
| Subcutaneous panniculitis-like T cell lymphoma |
| T-cell Lymphoblastic lymphoma |

Detailed entities of the 33 patients analyzed in our work arranged alphabetically
